# Exploring the Potential of Symmetric Exon Deletion to Treat Non-Ischemic Dilated Cardiomyopathy by Removing Frameshift Mutations in *TTN*

**DOI:** 10.3390/genes13061093

**Published:** 2022-06-19

**Authors:** Ignacio Rodriguez-Polo, Rüdiger Behr

**Affiliations:** 1 Research Platform Degenerative Diseases, German Primate Center—Leibniz Institute for Primate Research, 37077 Göttingen, Germany; rbehr@dpz.eu; 2 German Center for Cardiovascular Research (DZHK), Partner site Göttingen, 37077 Göttingen, Germany

**Keywords:** Titin, Obscurin, *TTN*, *OBSCN*, myoediting, exons, symmetry, CRISPR/Cas9

## Abstract

Non-ischemic dilated cardiomyopathy (DCM) is one of the most frequent pathologies requiring cardiac transplants. Even though the etiology of this disease is complex, frameshift mutations in the giant sarcomeric protein Titin could explain up to 25% of the familial and 18% of the sporadic cases of DCM. Many studies have shown the potential of genome editing using CRISPR/Cas9 to correct truncating mutations in sarcomeric proteins and have established the grounds for myoediting. However, these therapies are still in an immature state, with only few studies showing an efficient treatment of cardiac diseases. This publication hypothesizes that the Titin (*TTN*)-specific gene structure allows the application of myoediting approaches in a broad range of locations to reframe TTNtvvariants and to treat DCM patients. Additionally, to pave the way for the generation of efficient myoediting approaches for DCM, we screened and selected promising target locations in *TTN*. We conceptually explored the deletion of symmetric exons as a therapeutic approach to restore *TTN*’s reading frame in cases of frameshift mutations. We identified a set of 94 potential candidate exons of *TTN* that we consider particularly suitable for this therapeutic deletion. With this study, we aim to contribute to the development of new therapies to efficiently treat titinopathies and other diseases caused by mutations in genes encoding proteins with modular structures, e.g., Obscurin.

## 1. Introduction/Background

Inherited cardiomyopathies are the leading causes of cardiac-related deaths [1,2]. Dilated cardiomyopathy (DCM) is a disease that affects approximately 1 out of 2500 persons and has been found in the last few years with accelerated frequency [3,4,5]. DCM is diagnosed according to two criteria: (1) left ventricular enlargement, and (2) systolic dysfunction recognizable by the reduction in the myocardial contraction force [5]. DCM patients can display a broad range of phenotypes, ranging from heart failure to arrhythmias and thromboembolic disease [4]. Approximately 50% of the cases of DCM are inherited [6]. In familial dilated cardiomyopathy (familial DCM), structural or functional abnormalities develop due to a mutation, affecting the electrophysiological properties of the cardiomyocytes, e.g., calcium handling proteins, nuclear envelope proteins, or the contractile apparatus among others [1,3]. Mutations in more than 30 genes can lead to DCM, making it a highly complex and heterogeneous disease. A total of 25% of the cases of familial and 18% of sporadic DCM cases can be related to mutations (non-sense, frameshift, or essential splice site) of the sarcomeric protein Titin (*TTN*) [5,7,8,9,10]. Frameshift mutations in *TTN* alter the reading frame, leading to the premature termination of translation and thus generating truncated versions of the protein (TTNtv) [11,12].

Titin is a giant sarcomeric protein that can code up to 35,991aa (theoretical protein) (NCBI: NP_001254479) and spans half of the sarcomere [13]. The *TTN* gene encodes for the largest human protein and is composed of 364 exons, including a first non-coding exon. In humans, *TTN* is located on chromosome 2q31 [3]. TTN has a multitude of functions. It acts as a biological spring between the Z-disk and the M-line and serves as a scaffold of the sarcomere assembly. Additionally, it is a hot spot for protein–protein interactions, a key mediator of signal transduction in cardiomyocytes, and determines the passive tension of muscle fibers [8,11,12,14,15]. TTN regions are annotated according to their position in the sarcomere visualized by immune-electron micrographs, i.e., Z-disc, I-band, A-band, and M-line [16]. Titin undergoes extensive alternative splicing generating a plethora of isoforms. N2B and N2BA are the major cardiac isoforms and comprise the four regions: Z-line, I-band, A-band, and M-line [3,17]. The clinically most relevant mutations of *TTN* are located in the I-band and the A-band. This often leads to early stop codons and a mutated TTN lacking the C-terminal part of the A-band and M-line [12]. Additionally, TTNtv can lead to both dominant and recessive forms of cardiac and skeletal phenotypes depending on the nature of the mutation [15,18,19].

Many efforts have been made in the last few years to associate specific genotypic alterations with the phenotypic (symptomatic) spectrum of DCM [7,10,20,21]. Mapping the mutations that lead to TTNtv and ultimately to DCM is critical to generate efficient treatments [7,8,9,11].

To date, there are only limited therapeutic alternatives for DCM. In severe cases, heart transplantation is the only option. However, transplantation is a bottleneck due to the limited availability of donor hearts. In contrast, promising approaches to the treatment of a large number of DCM patients are genome editing technologies to restore the reading frame of *TTNtv* [4,11].

The CRISPR (Clustered regularly interspaced short palindromic repeats)/Cas system evolved as a powerful biotechnological tool to modify genomes in prokaryotes and eukaryotes [22,23,24]. Cas9 nucleases can generate double-strand breaks at specific sites in the genome. Cas9 is guided by short RNA guides (gRNA), derived from the CRISPR RNA array (crRNA) and trans-activating crRNA (tracrRNA) [22]. In contrast to other nucleases, such as transcription-activator-like effector nucleases (TALEN), zinc-finger nucleases (ZFN), or meganucleases, the CRISPR/Cas system is easy to apply and highly efficient, making it the method of choice for genome editing studies [25,26]. To date, CRISPR/Cas has had a strong impact on disease modeling and the understanding of biological mechanisms. A special focus is on the potential of CRISPR/Cas for in vivo genome editing [26,27,28,29,30].

A skip or deletion of in-frame mutated exons via the CRISPR/Cas9 system has long been considered a potential strategy to treat DCM and other cardiovascular diseases [25,31,32,33,34]. Recently, a variety of endonuclease-based experimental treatments were tested and established to overcome frameshift mutations in sarcomeric proteins [31,34,35,36]. These approaches can be categorized into three groups, i.e., (a) controlled splicing of mutated exons by inducing indel mutations in the splice acceptor–donor site, (b) full fragment/exon removal, and (c) restoration of the original wildtype sequence by targeting the mutated locus [31,37]. Approaches (a) and (b) lead to an incomplete protein; however, they have the potential to completely or partially restore its functionality [35,36]. In contrast, approach (c) restores the wildtype form of the protein [31]. In a recent study using patient-derived iPSC with a frameshift mutation in the A-band, the authors corrected the mutation by restoring the wildtype sequence using a Cas9 plus a gRNA targeting the mutated *TTN* allele and a single-stranded oligo as the donor for homology-directed repair. Furthermore, the iPSC-derived cardiomyocytes derived from the corrected cell line showed wildtype functionality as assessed by determining their force of contraction using engineered heart muscle (EHM) [38,39].

Besides endonuclease-based approaches, RNA-based therapeutics and splice-switching approaches have been tested to correct Titin, Dystrophin, and other sarcomeric protein frameshift mutations [40]. In Duchenne muscular dystrophy (DMD), mutations are concentrated in hotspots (exons 45 to 55 and exons 2 to 10). This allows a focus on specific gene locations to cover most of the mutations. In contrast, the mutations in *TTN* associated with severe DCM phenotypes are located in a wide range of sites and regions [10,11,15,40,41]. Recently, Gramlich et al. showed the beneficial potential of the *TTN* reframing strategy using antisense oligonucleotide (AON-) mediated exon skipping by correcting in vitro an autosomal dominant mutation in the giant *TTN* exon 326 [8]. Approaches relying on splice site mutations and genomic deletions always have to consider exon symmetry (exons whose number of base pairs is a multiple of 3 are symmetric). In order to repair the frameshift in an allele coding for a truncated version of a TTNtv protein (“reframing”), it is essential to select symmetric exons. The deletion of symmetric exons will not affect the reading frame of the wildtype protein but would restore a shortened version of the original open reading [42]. Aiming to contribute to the development of therapies to treat familial DCM, we evaluated the *TTN* structure from a therapeutic perspective.

## 2. The Hypothesis

We hypothesize that the Titin-specific gene structure allows the application of myo-editing approaches in a broad range of locations to reframe *TTNtv* variants and to treat DCM patients. Based on this, we selected exons that are highly accessible to recently developed genome editing tools to facilitate the establishment of novel CRISPR-based therapeutics to treat DCM.

**Figure 1:**
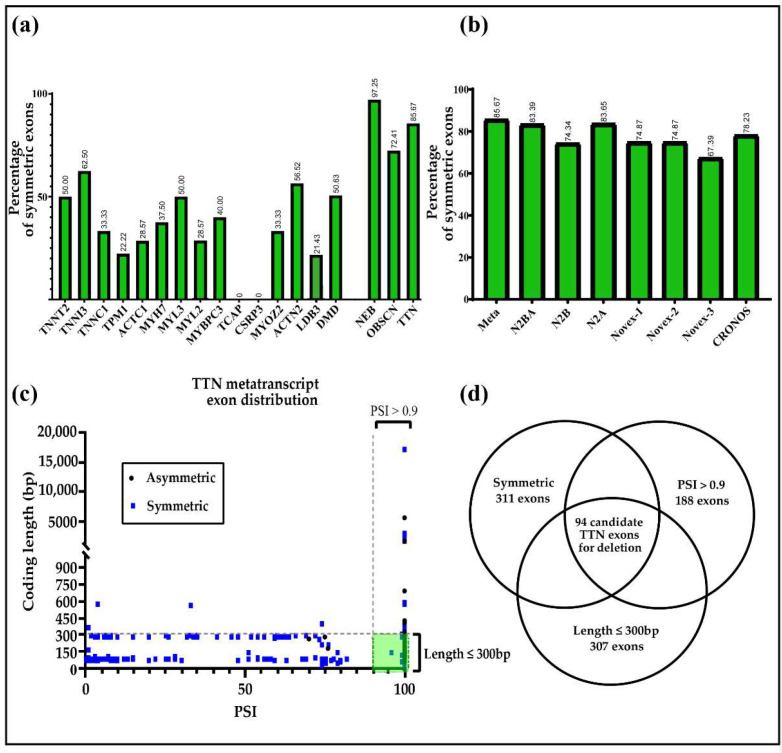
(**a**,**b**) Percentage of symmetric exons. (**a**) Percentage of protein-coding symmetric exons in different cardiac genes. *TNNT2* (8 symmetric exons/16 exons in total; 50%), *TNNI3* (5/8; 62.5%), *TNNC1* (2/6; 33.33%), *TPM1* (2/9; 22.22%), *ACTC1* (2/7; 28.57%), *MYH7* (15/40; 37.5%), *MYL3* (3/6; 50%), *MYL2* (2/7; 28.57%), *MYBPC3* (14/35; 40%), *TCAP* (0/2; 0%), *CSRP3* (0/7; 0%), *MYOZ2* (2/6; 33.33%), *ACTN2* (11/21; 52.38%), *LDB3* (3/14; 21.43%), *DMD* (40/79; 50.63%), *NEB* (177; 182; 97.25%), *OBSCN* (84/116; 72.41%), and *TTN* (311/363; 85.67%). (**b**) Percentage of symmetric protein-coding exons in the major *TTN* isoforms. Meta-transcript (311 symmetric exons/363-coding exons in total; 85.67%), N2BA (261/313; 83.38%), N2B (142/191; 74.34%), N2A (261/312; 83.65%), Novex-1 (143/191; 74.87%), Novex-2 (143/192; 74.86%), Novex-3 (30/46; 67.39%) and Cronos (97/124; 78.22%). Accession numbers of the different sequences can be found in (Tables S2 and S3). (**c**) Plot representing coding length versus the percentage of splice in of the different exons in *TTN* (ENST00000589042). Blue squares represent symmetric exons and black dots asymmetric exons. Limits of the exons’ size (of ≤300 bp) and PSI threshold of >0.9 are marked by dotted lines. The green square contains the exons highly suitable for removal according to the criteria set in this hypothesis. (**d**) Venn diagram of the parameters set for the identification of target exons. Selected exons are symmetric, have a high PSI (>0.9) value, and consist of 300 bp at a maximum.

The *TTN* gene has a very high percentage of symmetric exons compared to other cardiac-relevant genes. This fact provides a high number of potential target exons that can be deleted without altering the phase (Figure 1a). Additionally, the large number of fibronectin (FN-III) and immunoglobulin (Ig) repeated domains of TTN increase the probability of generating a functionally intact protein after the deletion of single symmetric exons (Table S1) [43,44].

Following this therapeutic strategy, the first step is the identification of the symmetric exons present in the gene (Figure 1a,b). However, to select candidate exons for deletion, some additional considerations must be taken into account. Targeted exons should be highly conserved amongst different isoforms, resulting in a high PSI (percentage of splice in) value. A PSI value of 1 indicates that an exon is constitutively present in all isoforms. A PSI value close to zero indicates that the respective exon is absent from most transcripts. Mutations located in *TTN* exons with a low PSI usually lead to mild phenotypes [9,11,44,45]. Finally, exon size also needs to be considered. The selection of small to medium-sized exons allows for deletion with a higher efficiency and prevents chromosomal alterations [46,47,48]. This will be particularly important regarding the (pre-) clinical translation of such therapies (Figure 1).

In accordance with the characteristics mentioned above, we selected a set of candidate exons for deletion from the *TTN* metatranscript (theoretical protein) (Table S1). We hypothesized that exons selected by (1) symmetry, (2) high conservation (PSI > 0.9), and (3) size of 300 bp or less would be particularly suitable for genomic exon deletion (Figure 1d).

## 3. The Titin Gene Contains a Large Number of Potential Target Exons

The *TTN* metatranscript sequence (ENST00000589042) was exemplarily evaluated in order to identify symmetric exons. Furthermore, the following major *TTN* isoforms were analyzed: N2BA (ENST00000591111), N2B (ENST00000460472), N2A (ENST00000342992), Novex-1 (ENST00000359218), Novex-2 (ENST00000342175), Novex-3 (ENST00000360870), and the recently discovered Cronos isoform (Figure 1b) [11,44,49]. The *TTN* metatranscript contains 85% (311/363) of symmetric protein coding exons, and the different isoforms range from 67 to 83% (Novex-3 and the long cardiac isoform N2BA, respectively) (Figure 1b, Table S3) [44].

To determine if the symmetric exon abundance was specific to *TTN* or if other relevant cardiac genes also showed this characteristic, we compared symmetric exon abundance in *TTN*, five thin filament proteins (*TNNT2*, *TNNI3*, *TNNC1*, *TPM1*, *ACTC1*), four thick filament proteins (*MYH7*, *MYL3*, *MYL2*, *MYBPC3*), six Z-disk proteins (*TCAP*, *CSRP3*, *MYOZ2*, *ACTN2*, *OBSCN*, *LDB3*), and the cytoskeletal protein Dystrophin (*DMD*). The analysis showed that except *TTN*, only Nebulin (*NEB*) and Obscurin (*OBSCN*) genes had an abundance of more than 73% symmetrical exons, while the statistical probability for the occurrence of symmetrical exons was ~33%. The remaining genes analyzed showed lower percentages of symmetric exons (Figure 1, Table S2).

After the identification of symmetric exons, we further categorized them according to their size. The CRISPR/Cas system has been tested in vitro for its efficiency to introduce small and large deletions [8,50]. These studies demonstrated a high variability of the efficiencies depending on the size of the removed DNA fragment, targeting location, and expression strategy. We selected exons that had 300 bp or less, given the increased efficiency in deletions with decreasing fragment size [46,47,48]. Furthermore, many exons in *TTN* are small to medium size (between 201 and 300 bp, 38%). The length of the majority of the *TTN* exons was ≤300 bp (307/363 exons, 84.56%) (Figure S1 and Figure 1c). This analysis of symmetry and size of the different exons indicated that *TTN* was particularly suitable for the proposed gene therapy approach by genomic exon deletion. The third factor taken into consideration was the level of conservation of the exons amongst the different isoforms. Only exons with a splice-in percentage higher than 0.9 (PSI > 0.9) were taken into account. In conclusion, candidate exons for therapeutic deletion were chosen according to size, symmetry, and PSI, leading to a set of 94 candidate exons (Table S1, Figure 1d).

In addition, one additional consideration that must be taken into account to select exons suitable for therapeutic deletion is the predicted function of the corresponding protein domain. We categorized the domains associated with the selected 94 candidate exons for deletion (Table S1). TTN is constituted mainly by repeated FN-III and IgI domains, whose abundance is also reflected by the selected candidate exons for deletion (Table S1). Proteins with a highly repetitive structure are more likely to conserve their integrity if one of the exons coding for repeated domains is deleted. Additionally, by characterizing the corresponding protein domains of the selected exons with tools such as DIGGER, it is possible to (at least partially) predict the alterations in the TTN interactome resulting from the deletion of specific exons. For instance, we exemplarily evaluated the impact of the deletion exons 14 and 231, respectively (Table S4) [51]. Although it is possible to obtain in silico information regarding the potential alterations in the interaction of TTN with its partners, it is important to be cautious since these are only predictions, and the in silico analysis needs to be proven experimentally (check Section 4, Evaluation of the Hypothesis).

In our analysis (Figure 1a), the direct comparison of symmetric exon abundance of *TTN* compared with other cardiac relevant proteins revealed that *OBSCN* also contained a high number (and high percentage) of symmetric exons compared to the rest of the analyzed genes (Figure 1a). Additionally, this sarcomeric protein shared most of the characteristics that make *TTN* an interesting candidate for the systematic exploration of symmetric exon deletion. This includes length, highly repeated Ig and FN-III domains, and most importantly, biomedical relevance, e.g., mutations in both TTN and OBSCN have been linked with hypertrophic cardiomyopathy [52,53,54]. However, even though there is some evidence [54,55], the extent of the involvement of mutations in *OBSCN* in the development of heart disease has not finally been determined [56]. We applied a homologous strategy for *TTN* to select *OBSCN* exons according to symmetry, size, and conservation (Figure S2). For symmetry and size, the same parameters applied to select *TTN* exons were considered. However, we based our selection of highly conserved exons on the known biological functions of *OBSCN* isoforms. *OBSCN* contains several splice variants, including two large main isoforms A (~720 kDa) and B (~870 kDa) (Figure S2a, Table S5). Therefore we selected exons conserved between both isoforms (Figure S2b) [53]. After analysis, 59 *OBSCN* candidate exons were identified for potential therapeutic removal (Table S6).

Together with TTN and OBSCN, Nebulin (NEB) completes the family of three members of giant sarcomeric proteins present in striated muscles. Additionally, *NEB* also presents a high percentage of symmetric exons (97.25% in the metatranscrip and 97.79% in the two major isoforms S21a and S21b) [57]. Furthermore, mutations in Nebulette, a member of the Nebulin family, have been demonstrated to be involved in the development of different cardiomyopathies [53,58,59,60]. However, the structure of *NEB* results from extensive duplications leading to a gene mainly composed of tandem repeats constituting the middle part of the gene as super-repeated areas. This is a clear difference compared with *TTN* and *OBSCN* [53]. This has implications for myoediting since the flexibility and specificity of targeting these areas with genome editing tools is limited. Therefore reframing strategies for *NEB* deserve a separate approach; hence, this gene was not considered in detail in this manuscript [53].

## 4. Evaluation of the Hypothesis

To address the potential for translation to the clinics, the safety and efficacy of this approach have to be evaluated. Each of the 94 proposed *TTN* locations needs to be tested individually to clarify gRNA efficiency for the deletions and possible adverse effects caused by the resulting gene versions lacking specifically deleted exons [30]. Additionally, the selected locations have to be related to mutations found in DCM patients.

The first step is to generate data for the in vitro validation of the system. This can be completed in patient-specific induced pluripotent stem cell-derived cardiomyocytes (piPSC-CM) [32,61,62,63]. To test the safety of the deletion, adverse effects derived from the exon removal need to be evaluated. Once the treatment has been validated in vitro, in vivo testing using different animal models will be required in an evolutionary bottom-up approach using, e.g., zebrafish, mouse or rat, and for final preclinical testing non-human primates [32].

**Figure 2:**
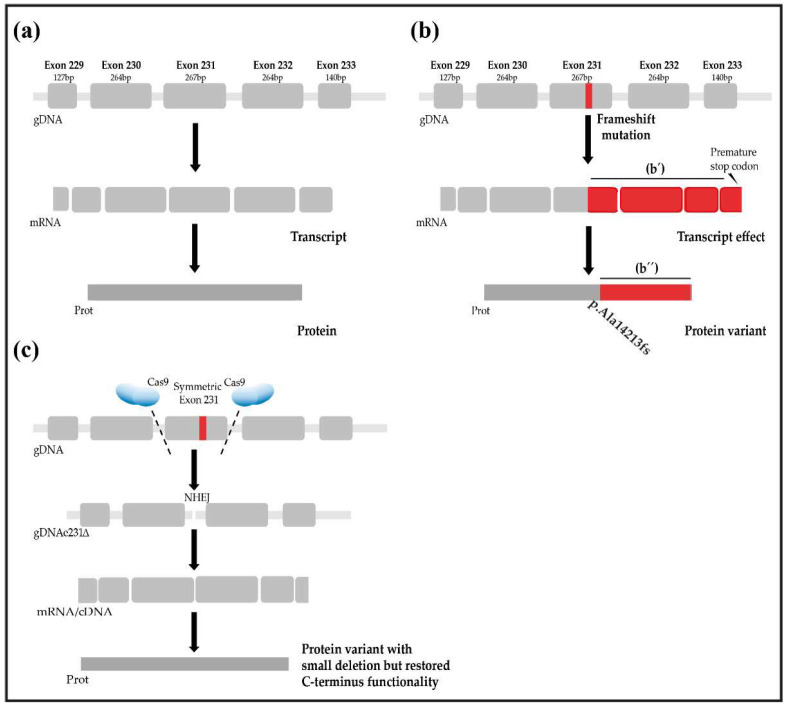
(**a**–**c**) Overview of the exon deletion approach to restore functional Titin. (**a**) Schematic representation of the canonical transcription and translation of *TTN*. (**b**) The functional protein is truncated by a point mutation (frameshift). *TTNtv* leads to (b’) a shifted reading frame with a premature stop codon and ultimately to a protein with (b”) a non-sense C-terminus. Exemplarily presented with a frameshift mutation in exon 231 of *TTN* that leads to a truncated and partially non-sense version of the protein. The red color indicates a non-sense amino acid sequence different from TTN that originates from the frameshift mutation. The frameshift causes, at the same time, the introduction of a stop codon on the transcript level, which in turn results in a truncated protein variant. (**c**) Symmetric exon deletion to restore TTN functionality. Symmetric exons with mutations are deleted with a system combining Cas9 plus two gRNAs, targeting intronic sites up- and down-stream of the candidate exon. TTN expression results in a minimally shorter but putatively functional protein. Schematic representation of the deletion of a symmetric exon to remove the frameshift mutation located in exon 231. Double strand breaks in *TTN* are repaired after CRISPR/Cas9 editing without the need for a homologous template by non-homologous end joining (NHEJ).

**Figure 3:**
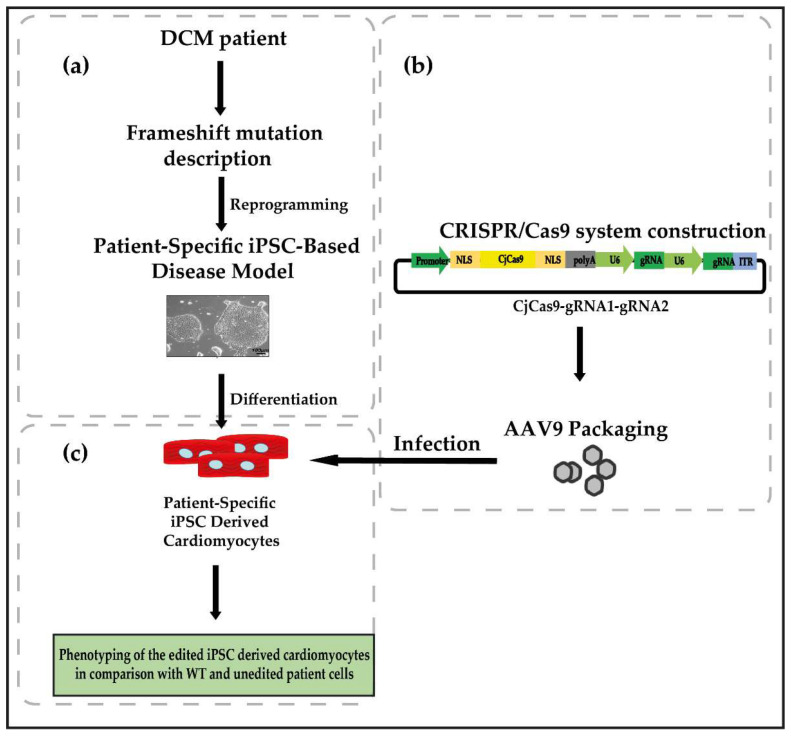
Flowchart of the in vitro testing and phenotyping of the exon deletion system to restore TTN functionality in DCM patients. (**a**) Validation of the system will be performed using DCM patient-specific iPSC-derived cardiomyocytes. (**b**) AAV expression vector containing, e.g., Cas9 ortholog, *Campylobacter jejuni* Cas9 plus two gRNA to target sequences up- and downstream of the mutated target exon. The vector will be packaged in AAVs with tropism for cardiomyocytes (CM). (**c**) After the successful delivery of the CRISPR/Cas system with two gRNAs by infection, detailed evaluation of the safety and efficacy of the system will be performed by in vitro phenotyping of the treated iPSC-derived CM.

The delivery of the CRISPR system in vivo is one of the major limitations of this approach and for genome editing therapies of the heart in general [27,64]. AAV-based therapies present a promising approach for gene-specific editing. Improvements in tissue- and cell type-specific targeting of AAV serotypes enhance the specificity of the treatment, thereby reducing off-target effects [27,65]. The traditional and recently engineered AAV serotypes for tissue-targeted delivery in combination with minimal tissue-specific promoters and specific administration routes for the virus make this system ideal for the delivery of CRISPR/Cas [64,66]. Although AAVs are an attractive vehicle, their relatively small viral genome and, therefore, reduced packaging capacity limits the delivery of large transgenes. This has to be considered, especially when attempting to package Cas9 plus the two gRNAs (Figure 2 and Figure 3). Using smaller Cas9 orthologues, in comparison with the most broadly used spCas9 (*Streptococcus pyogenes*-derived Cas9 (SpCas9) (4.1 kb)) can help to overcome this problem. *Campylobacter jejuni*-derived Cas9 (CjCas9) (984 amino acids, 2.95 kb) or *Staphylococcus aureus*-derived Cas9 (SaCas9) (1053 amino acids, 3.16 kb), are both validated endonucleases that can be used [66,67,68]. Additional space can be saved during the vector construction using truncated regulatory elements such as the promoter driving the expression of the Cas9 (miCMV/H1 promoter) or the polyadenylation signal [27]. Several AAV serotypes have been tested in different animal and human models. AAV8 and AAV9 have been shown to infect the heart efficiently [27,69,70] (Figure 3). Using the mentioned serotypes together with a suitable administration route may eventually lead to the efficient and specific targeting of specific *TTN* exons in human cardiomyocytes in vivo.

## 5. Consequences of the Hypothesis and Discussion

TTN plays a key role in striated and cardiac muscle cells [9,12,14]. Frameshift mutations in this giant sarcomeric protein lead to several diseases occurring due to alterations in the biological function [10,12]. This includes non-ischemic DCM, which is the most common form of cardiomyopathy and originates in 25% of the cases from truncation mutations in *TTN* [3,10,43]. Dissecting the pathological phenotype derived from the different truncation mutations is challenging, although the introduction of novel sequencing technologies, e.g., next-generation sequencing (NGS), has provided a new perspective on *TTN* mutation studies [3,44,49]. Current therapies to overcome non-ischemic DCM are based on treatments to inhibit the enlargement of the heart chamber in patients where the pathology has not fully developed. Antiarrhythmics and drugs to decrease blood pressure are usually the treatments of choice. Considering limited therapies and the lack of hearts available for transplantation, the development of novel therapies is crucial. We and others think that myoediting could be a solution to treat a substantial number of inherited and sporadic DCM cases. CRISPR-based approaches have previously demonstrated their applicability in sarcomeric protein mutation correction in vitro and in vivo [8,27,35,36,40].

Here, we hypothesized that the *TTN* gene structure allows the application of myo-editing approaches in a broad range of locations to reframe TTNtv variants (Figure 1) [44]. In order to further substantiate our hypothesis, we generated a list of candidate sites that could be promising for these therapies. Additionally, the *OBSCN* gene shares key characteristics with *TTN*, including a high abundance of symmetric exons. Therefore, we also considered this gene in our analysis. The illustrated approach has already been tested in other sarcomeric proteins [1]; however, to our knowledge, it has never been applied to *TTN*, or *OBSCN* [33]. We identified a set of candidate exons that fulfilled the requirements of (1) symmetry, (2) high conservation, and (3) size of ≤300 bp (Tables S1 and S6, Figure 1 and Figure S2). Symmetric exon deletion will conserve (or restore) the reading frame and, therefore, the general integrity of the transcript. Mutations in exons not conserved between the different isoforms must be interpreted with caution because they may have little or no effect on the resulting populations of TTN proteins within a specific cell [11,21,41]. Therefore, we only considered exons highly conserved in the various transcripts. Additionally, filtering the exons by size is critical as an inverse correlation between exon size and deletion efficiency has been shown [46,47,48]. This is a critical point regarding the translation of the therapy to the clinics [37,46,48,71]. The *TTN* gene contains 94 candidate exons that fulfill the criteria set in the present study, while *OBSCN* contains 59 such exons. Therefore, *TTN* and *OBSCN* are promising candidate genes with a high clinical relevance for the proposed type of gene therapy [13]. Additionally, using the information provided in this study, it is possible to predict in silico the impact on the protein functionality of the deletion of specific exons of *TTN* or *OBSCN* (Tables S1, S4 and S6).

Full exon deletion as therapy for average-sized genes has limited applicability (the average number of exons per gene in the human genome is 8.8) [42,72]. The biological function of proteins coded by a few exons will probably only be severely affected by the removal of one exon. In the case of giant sarcomeric proteins, these therapies can be applied with more confidence and in a broader range of locations due to the modular structure of the encoded proteins (Tables S1 and S6). This increases the likelihood that the protein keeps its biological function after the removal of a single exon [73]. Other authors have already successfully tested the Cas9 protein plus two guide systems for reframing other striated and cardiac muscle proteins [8,74].

We are aware that the application of the suggested therapy has to be carefully evaluated in each particular case. (1) It is necessary to continue assessing the feasibility and safety of the CRISPR system to efficiently delete full exons in vivo [24,30,32,66,68,75]. (2) The functionality of the protein has to be evaluated after exon deletion. Specifically, deleted exons could play a crucial role in the biophysical properties of the protein. (3) It is necessary to design highly efficient and selective delivery systems, e.g., AAVs.

Besides the specific tropism and moderate immunogenicity, another advantage of AAV delivery is the loss of the viral vectors over time. This particularity could be an advantage to achieve (desirable) transient CRISPR expression and reduce the possibility of off-targets and chromosomal abnormalities [25,27,65,76,77].

Additionally, it is important to evaluate the suggested therapy in relation to the different patient genotypes and phenotypes. For example, some forms of skeletal and cardiac titinopathies are associated with recessive *TTNtv* and do not show phenotypic alterations unless they are associated with another mutation [78,79]. In those cases, predicting the therapeutic potential might be challenging. Each case would require an individual assessment since one single *TTNtv* can derive a broad spectrum of phenotypes depending on the associated mutation [15].

For the in vitro validation of the approach, it is important to consider that many DCM phenotypes cannot be fully recapitulated in 2D cultures or in immature iPSC-CM. Therefore, 3D cardiomyocyte cultures giving rise to myocardium-like structures can better mimic DCM and validate possible phenotype rescue [12,31,39,63,80]. Additionally, the maturation of the iPSC-CMs can be also increased by controlling the stiffness of the culture substrates [81], or scaffolds [82], among others [7].

Even though the efforts that are yet to be made in order to optimize the approach stated here are laborious, the outcomes will increase our understanding of the mutations underlying DCM. The insights gathered from in vitro and in vivo experiments might lead to the efficient treatment of at least a few of the proposed mutation sites. Moreover, it is important to consider that the same validated CRISPR system could be used to treat different mutations in the same exon. Additionally, we believe that with the establishment and application of the proposed therapy approach, not only could DCM be treated, but given the number of *TTN*-dependent myopathies, the reframing system could also be translated to those pathologies [83]. For instance, other cardiac and skeletal muscle caused by *TTNtv* could be addressed by the exon deletion approach [15,18,79]. This highlights the broader applicability of the proposed approach to genetic diseases affecting a broader range of patients [43].

Another application of this approach is in the investigation of the correlation between the position of a mutation and the severity of the DCM phenotype. A variety of studies have focused on finding the relationship between the severity of DCM and the position of the mutation, focusing on (1) alternative splicing, (2) the alternative Cronos promoter (3), or the proximity to the C-terminus [12,20,45]. In a recent study McAfee et al., showed the presence of *TTNtv* in DCM patients and how this comes together with a reduced amount of full TTN in DCM hearts. This study supported the view that both dominant-negative forms of TTNtv and the haploinsufficiency of *TTN*, together with additional risk factors, contribute to the development of DCM and eventually lead to DCM in patients bearing truncations [45]. These findings were supported by a parallel study by Fomin and colleagues [38]. Deleting the proposed exons could further help to clarify how the mutations in the different parts of *TTN* lead to the different DCM phenotypes, discriminating between frameshift and deletion effects. If these experiments can explain the correlation between pheno- and genotype, these insights may facilitate prophylactic treatments. This reframing strategy may, in the future, be applied to affected infants or even in the fetus in order to prevent developmental heart defects, which eventually may lead to functional impairments [43].

Available patient data shows that DCM-causing mutations are not solely located in exons investigated in this study, e.g., exon 326. For this reason, we believe that it is necessary to combine a broader panel of therapeutic approaches to address all different sites of a gene. In the case of *TTN* exon 326, its size may exclude efficient deletion. Other approaches such as targeting the splice acceptor–donor site or the recently emerging base editing technology need to be considered in these cases [12].

In conclusion, we propose that the complete deletion of selected symmetric exons in *TTN* and other giant sarcomeric proteins could be used as a therapy to overcome a subset of cases of familial DCM with severe phenotypes. Additionally, we also propose a list of potential exons suitable to test this approach. We are already testing the deletion of some of the selected exons in vitro using human iPSCs. In conclusion, we believe that exon deletion approaches can expand the currently still experimental toolbox of approaches to efficiently treat sarcomeric protein-related heart diseases.

## Supplementary Materials

The following supporting information can be downloaded at: https://www.mdpi.com/article/10.3390/genes13061093/s1, Table S1: List of *TTN* candidate exons for therapeutic removal; Table S2: Quantification of symmetric exons in selected cardiac relevant genes; Table S3: Symmetric exon quantification in the major *TTN* isoforms; Figure S1: Distribution of *TTN* exons according to their size; Table S4: Predicted alterations in TTN interactions with partner proteins result from candidate exon deletion; Figure S2: *OBSCN* exon analysis for therapeutic removal; Table S5: Symmetric exon quantification in the major *OBSCN* isoforms. Table S6: *OBSCN* list of candidate exons for removal.
