# Supplementary material for: Exploring the Potential of Symmetric Exon Deletion to Treat Non-Ischemic Dilated Cardiomyopathy by Removing Frameshift Mutations in TTN
Source: Genes (Basel). 2022 Jun 19;13(6):1093. doi: 10.3390/genes13061093 (PMC9222585; doi:10.3390/genes13061093)
Supplement: Supplementary file 1 [file genes-13-01093-s001.zip › genes-1681912-supplementary.pdf]

Supplementary material

Table S1. List of candidate exons for therapeutic removal. Exons were selected from the *TTN* metatranscript (ENST00000589042) and filtered according to symmetry, conservation between the different isoforms (PSI > 0.9), and size (≤ 300bp). A comprehensive analysis of Titin exons can be found in [44]. Additionally, domains and domain identifications (Pfam ID) are included in the table, together with a prediction of the number of interactions mediated by each domain (domain interactions, DI), predicted using DIGGER [51].

Exon Nº	Exon ID	Length	Location	Domains	Exon Phase	Exon PSI	Pfam ID	DI
**3**	ENSE00003807606	204	Z-disk	Ig-like 1	1	1	PF07679	23
**4**	ENSE00003810976	288	Z-disk	Ig-like 2	1	1	PF07679	23
**8**	ENSE00003803639	153	Z-disk	Z-repeat 1	0	1	PF09042	1
**9**	ENSE00003810843	138	Z-disk	Z-repeat 2	0	1	PF09042	1
**10**	ENSE00003803899	126	Z-disk	Z-repeat 3	0	1	PF09042	1
**13**	ENSE00003805623	138	Z-disk	Z-repeat 6	0	0,96	PF09042	1
**14**	ENSE00003801659	294	Z-disk	Z-repeat 7	0	1	PF09042	1
**15**	ENSE00003804455	123	Z-disk		0	0,99		
**16**	ENSE00003810306	282	near Z-disk		0	0,99		
**17**	ENSE00003805296	66	near Z-disk		0	0,99		
**20**	ENSE00003804962	216	near Z-disk	Ig-like 4	2	1	PF07679	23
**23**	ENSE00003807963	234	near Z-disk	Ig-like 5	0	1	PF07679	23
**26**	ENSE00003811446	165	near Z-disk	Ig-like 6	1	1	PF07679	23
**29**	ENSE00003809440	282	I-band	Ig-like 11	1	1	PF07679	23
**30**	ENSE00003802960	267	I-band	Ig-like 12	1	1	PF07679	23
**31**	ENSE00003804241	273	I-band	Ig-like 13	1	1	PF07679	23
**32**	ENSE00003806988	264	I-band	Ig-like 14	1	1	PF07679	23
**33**	ENSE00003804968	261	I-band		1	1	PF07679	23
**34**	ENSE00003809850	261	I-band	Ig-like 15	1	1	PF07679	23
**35**	ENSE00003806479	264	I-band		1	1	PF07679	23
**36**	ENSE00003804523	261	I-band		1	1	PF07679	23
**37**	ENSE00003807378	261	I-band	Ig-like 16	1	1	PF07679	23
**38**	ENSE00003807271	261	I-band	Ig-like 17	1	1	PF07679	23
**42**	ENSE00003802092	285	I-band	Ig-like 19	1	1	PF07679	23
**43**	ENSE00003807103	126	I-band	Ig-like 20	1	1	PF07679	23
**44**	ENSE00003801355	189	I-band	Ig-like 20	1	1	PF07679	23
**47**	ENSE00003810791	57	I-band		1	1		
**49**	ENSE00003805227	279	I-band	Ig-like 24	1	1	PF07679	23
**219**	ENSE00003804802	81	I-band	PEVK27	1	1		
**220**	ENSE00003811452	75	I-band	PEVK28	1	1		
**221**	ENSE00003807205	90	I-band		1	1		
**222**	ENSE00003806747	63	I-band	PEVK29	1	1		
**223**	ENSE00003810755	90	I-band	PEVK30	1	1		
**226**	ENSE00003803996	279	I-band	Ig-like 81	1	1		
**227**	ENSE00003807976	276	I-band	Ig-like 82	1	1		
**230**	ENSE00003811410	264	I-band		1	1	PF07679	23
**231**	ENSE00003805150	267	I-band	Ig-like 83	1	1	PF07679	23
**232**	ENSE00003801907	264	I-band	Ig-like 84	1	1	PF07679	23
**235**	ENSE00003811384	267	I-band	Ig-like 85	1	1	PF07679	23
**236**	ENSE00003801954	267	I-band		1	1	PF07679	23

**237**	ENSE00003809996	267	I-band	Ig-like 86	1	1	PF07679	23
**245**	ENSE00003811387	267	I-band	Ig-like 90	1	1	PF07679	23
**246**	ENSE00003802653	267	I-band	Ig-like 91	1	1	PF07679	23
**247**	ENSE00003802540	279	I-band	Ig-like 92	1	1	PF07679	23
**250**	ENSE00003807647	267	I-band		1	1	PF07679	23
**251**	ENSE00003802353	270	I-band	Ig-like 95	1	1	PF07679	23
**256**	ENSE00003809985	285	A-band		1	1	PF07679	23
**261**	ENSE00003809522	288	A-band	Ig-like 96	1	1	PF07679	23
**262**	ENSE00003804879	297	A-band	Fibronectin type-III 6	1	1	PF00041	26
**265**	ENSE00003806136	300	A-band	Fibronectin type-III 8	1	1	PF00041	26
**266**	ENSE00003804290	300	A-band	Fibronectin type-III 9	1	1	PF00041	26
**270**	ENSE00003808061	279	A-band	Ig-like 97	1	1	PF07679	23
**271**	ENSE00003811164	300	A-band	Fibronectin type-III 12	1	1	PF00041	26
**275**	ENSE00003809346	300	A-band	Fibronectin type-III 15	1	1	PF00041	26
**276**	ENSE00003806782	297	A-band	Fibronectin type-III 16	1	1	PF00041	26
**277**	ENSE00003804121	285	A-band	Ig-like 99	1	1	PF07679	23
**278**	ENSE00003804454	294	A-band	Fibronectin type-III 17	1	1	PF00041	26
**279**	ENSE00003803420	300	A-band	Fibronectin type-III 18	1	1	PF00041	26
**285**	ENSE00003801625	33	A-band	Ig-like 101	0	1	PF07679	23
**287**	ENSE00003804239	300	A-band	Fibronectin type-III 22	1	1	PF00041	26
**289**	ENSE00003801391	297	A-band	Ig-like 102	1	1	PF07679	23
**290**	ENSE00003806021	300	A-band	Fibronectin type-III 24	1	1	PF00041	26
**294**	ENSE00003808790	282	A-band	Ig-like 103	1	1	PF07679	23
**297**	ENSE00003807039	282	A-band	Ig-like 104	1	1	PF07679	23
**298**	ENSE00003804106	300	A-band	Fibronectin type-III 29	1	1	PF00041	26
**301**	ENSE00003807476	282	A-band	Ig-like 105	1	1	PF07679	23
**302**	ENSE00003807363	300	A-band	Fibronectin type-III 32	1	1	PF00041	26
**303**	ENSE00003808994	294	A-band	Fibronectin type-III 33	1	1	PF00041	26
**306**	ENSE00003805661	285	A-band	Ig-like 109	1	1	PF07679	23
**307**	ENSE00003804742	300	A-band	Fibronectin type-III 42	1	1	PF00041	26
**309**	ENSE00003801707	276	A-band	Ig-like 110	1	1	PF07679	23
**310**	ENSE00003810399	300	A-band	Fibronectin type-III 44	1	1	PF00041	26
**312**	ENSE00003802505	300	A-band	Fibronectin type-III 46	1	1	PF00041	26
**313**	ENSE00003806397	288	A-band	Ig-like 111	1	1	PF07679	23
**314**	ENSE00003807889	297	A-band	Fibronectin type-III 47	1	1	PF00041	26
**317**	ENSE00003807542	288	A-band		1	1	PF07679	23
**318**	ENSE00003810775	291	A-band	Fibronectin type-III 50	1	1	PF00041	26
**319**	ENSE00003811210	288	A-band	Fibronectin type-III 51	1	1	PF00041	26
**321**	ENSE00003808777	105	A-band	Fibronectin type-III 52	1	1	PF00041	26
**322**	ENSE00003806939	198	A-band	Fibronectin type-III 53	1	1	PF00041	26
**323**	ENSE00003811024	297	A-band	Fibronectin type-III 54	1	1	PF00041	26
**327**	ENSE00003802597	297	A-band	Fibronectin type-III 99	1	1	PF00041	26
**330**	ENSE00003806587	297	A-band	Fibronectin type-III 102	1	1	PF00041	26
**331**	ENSE00003807681	288	A-band	Ig-like 130	1	1	PF07679	23
**332**	ENSE00003811102	300	A-band	Fibronectin type-III 103	1	1	PF00041	26
**336**	ENSE00003811205	294	A-band	Fibronectin type-III 110	1	1	PF00041	26
**337**	ENSE00003808021	288	A-band	Ig-like 133	1	1	PF07679	23
**338**	ENSE00003804355	300	A-band	Fibronectin type-III 111	1	1	PF00041	26
**342**	ENSE00003801213	291	A-band	Ig-like 136	1	1	PF07679	23

**343**	ENSE00003809128	297	A-band	Fibronectin type-III 119	1	1	PF00041	26
**346**	ENSE00003809946	282	A-band	Ig-like 137	1	1	PF07679	23
**348**	ENSE00003802641	288	A-band	Ig-like 138	1	1	PF07679	23
**349**	ENSE00003806194	300	A-band	Fibronectin type-III 124	1	1	PF00041	26
**354**	ENSE00003810204	300	A-band	Fibronectin type-III 129	1	1	PF00041	26

Table S2. Quantification of symmetric exons in selected cardiac relevant genes (*Homo sapiens*). Cardiac troponin T (*TNNT2*), Cardiac troponin I (*TNNI3*), Cardiac troponin C (*TNNC1*), α-Tropomyosin (*TPM1*), α-Cardiac actin (*ACTC1*), β-Myosin heavy chain (*MYH7*), Ventricular regulatory light chain (*MYL3*), Ventricular essential light chain (*MYL2*), Cardiac myosin-binding protein C (*MYBPC3*), Titin (*TTN*), T-cap (*TCAP*), MLP (*CSRP3*), Myozenin-2 (*MYOZ2*), α-Actinin (*ACTN2*), Obscurin (*OBSCN*), Cypher (*LDB3*), and Dystrophin (*DMD*).

	**Gene**	**No. Exons***	**Accession Number**	**No. Symmetric Exons**	**Percentage of Symmetric Exons (%)**
Thin filament proteins	*TNNT2*	16	ENST00000509001.5	8	50
	TNNI3	8	ENST00000665070.1	5	62,5
	TNNC1	6	ENST00000232975.7	2	33,33
	TPM1	9	ENST00000358278.7	2	22,22
	ACTC1	7	ENST00000290378.6	2	28,57
Thick filament proteins	*MYH7*	40	ENST00000355349.4	15	37,5
	MYL3	6	ENST00000395869.5	3	50
	MYL2	7	ENST00000228841.15	2	28,57
	MYBPC3	35	ENST00000545968.6	14	40
Titin and Z-disc proteins	*TTN*	363	ENST00000589042.5	311	85,67
	TCAP	2	ENST00000309889.3	0	0
	CSRP3	7	ENST00000533783.2	2	28,57
	MYOZ2	6	ENST00000307128.6	2	33,33
	ACTN2	21	ENST00000366578.6	11	52,38
	OBSCN	116	ENST00000570156.7	85	73,28
	LDB3	14	ENST00000429277.7	3	21,43
Cytoplasmic protein	*DMD*	79	ENST00000357033.9	40	50,63

*Number of protein coding exons.

Table S3. Symmetric exon quantification in the major *TTN* isoforms (*Homo sapiens*).

Transcript	No. Exons	Accession Number	No. Symmetric Exons	Percentage of Symmetric Exons (%)
Meta	363	ENST00000589042.5	311	85,67
N2BA	313	ENST00000591111.5	261	83,39
N2B	191	ENST00000460472.6	142	74,35
N2A	312	ENST00000342992.10	261	83,65
Novex-1	191	ENST00000359218.10	143	74,87
Novex-2	191	ENST00000342175.11	143	74,87
Novex-3	46	ENST00000360870.10	30	65,22
CRONOS	124		97	78,23

Figure S1

(a)

	Exon size (bp)				
	**<100**	**100 -200**	**201 -300**	**301 -400**	**>400**
Nº of exons	116	50	141	35	21
Percentage (%)	31.95	13.77	38.84	9.64	5.78

(b)

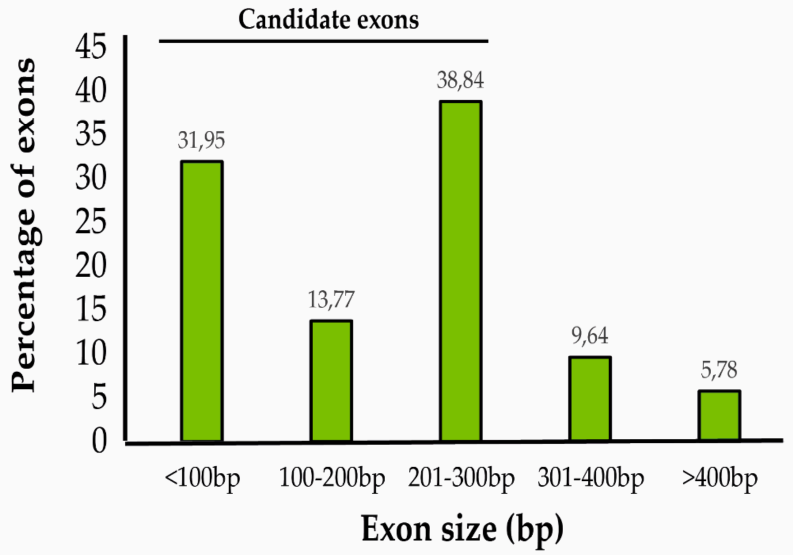

Figure S1. (**a**)(**b**) Distribution of *TTN* exons according to their size, in base pairs (bp). Defined size rages, smaller than 100bp (< 100), between 100 and 200bp (100–200), between 201 and 300bp (201–300), between 301 and 400b'p (301–400), and bigger than 400 bp (> 400).

Table S4. Predicted alterations in TTN interactions with partner proteins result from candidate exon deletion. Exemplarily shown for exon 14 (ENSE00003801659) (left table) or exon 231 (ENSE00003805150) (right table). The table lists the different interactions of a specific domain constituted by each respective exon, 14 or 231. Additionally, it is predicted if the interaction is conserved after exon deletion (Retained/Affected/Missing). The score is based on the percentage of deleted interacting domains with a specific partner protein, e.g., if two TTN domains mediate the interaction with one protein and one of them is deleted, the score will be 0,5. In contrast, if the interaction is mediated only by one domain and this is the one deleted, the score will be cero, showing the loss of that interaction. Analysis was performed using DIGGER [51].

Deletion of Exon 14						Deletion of Exon 231					
nº	Affected Protein	Partner Protein	NCBI Gene ID	Protein-Protein Interaction	Score	nº	Affected Protein	Partner Protein	NCBI Gene ID	Protein-Protein Interaction	Score
1	TTN	CAPN3	825	Retained	1.00	1	TTN	CAPN3	825	Retained	1.00
2	TTN	DYRK2	8445	Retained	1.00	2	TTN	NEDD8	4738	Retained	1.00
3	TTN	SRSF2	6427	Retained	1.00	3	TTN	HEY1	23462	Retained	1.00
4	TTN	CUL3	8452	Retained	1.00	4	TTN	CPSF6	11052	Retained	1.00
5	TTN	RBM3	5935	Retained	1.00	5	TTN	SP1	6667	Retained	1.00
6	TTN	CUL7	9820	Retained	1.00	6	TTN	PUF60	22827	Retained	1.00
7	TTN	EGFR	1956	Retained	1.00	7	TTN	EGFR	1956	Retained	1.00
8	TTN	PUF60	22827	Retained	1.00	8	TTN	CUL7	9820	Retained	1.00
9	TTN	MYBPC3	4607	Retained	1.00	9	TTN	RBM3	5935	Retained	1.00
10	TTN	CHEK1	1111	Retained	1.00	10	TTN	CUL3	8452	Retained	1.00
						11	TTN	SRSF2	6427	Retained	1.00
						12	TTN	BRCA1	672	Retained	1.00
						13	TTN	SPEN	23013	Retained	1.00
						14	TTN	TLE3	7090	Retained	1.00
						15	TTN	CALM1	801	Retained	1.00
						16	TTN	ACTN1	87	Retained	1.00
						17	TTN	NBR1	4077	Retained	1.00
						18	TTN	TOP2A	7153	Retained	1.00
						19	TTN	FHL1	2273	Retained	1.00
						20	TTN	CUL4B	8450	Retained	1.00
						21	TTN	VAV2	7410	Retained	1.00
						22	TTN	SQSTM1	8878	Retained	1.00
						23	TTN	YWHAQ	10971	Retained	1.00
						24	TTN	RAPGEF2	9693	Retained	1.00
						25	TTN	FHL2	2274	Retained	1.00
						26	TTN	CUL2	8453	Retained	1.00
						27	TTN	KRAS	3845	Retained	1.00
						28	TTN	AGRN	375790	Retained	1.00
						29	TTN	EWSR1	2130	Retained	1.00
						30	TTN	BIRC3	330	Retained	1.00
						31	TTN	CHEK2	11200	Affected	0.80
						32	TTN	OBSCN	84033	Affected	0.72
						33	TTN	MYBPC3	4607	Affected	0.67
						34	TTN	CHEK1	1111	Affected	0.67
						35	TTN	ANK1	286	Affected	0.67
						36	TTN	SRPK2	6733	Affected	0.67
						37	TTN	MYPN	84665	Affected	0.67
						38	TTN	DYRK2	8445	Affected	0.67
						39	TTN	WEE1	7465	Affected	0.67
						40	TTN	MAP2K1	5604	Affected	0.67
						41	TTN	CDK2	1017	Affected	0.67
						42	TTN	MAPK1	5594	Affected	0.67
						43	TTN	ANKRD23	200539	Affected	0.50
						44	TTN	ANKRD2	26287	Affected	0.50
						45	TTN	ANKRD1	27063	Affected	0.50
						46	TTN	NTRK1	4914	Affected	0.50
						47	TTN	TCAP	8557	Missing	0.00

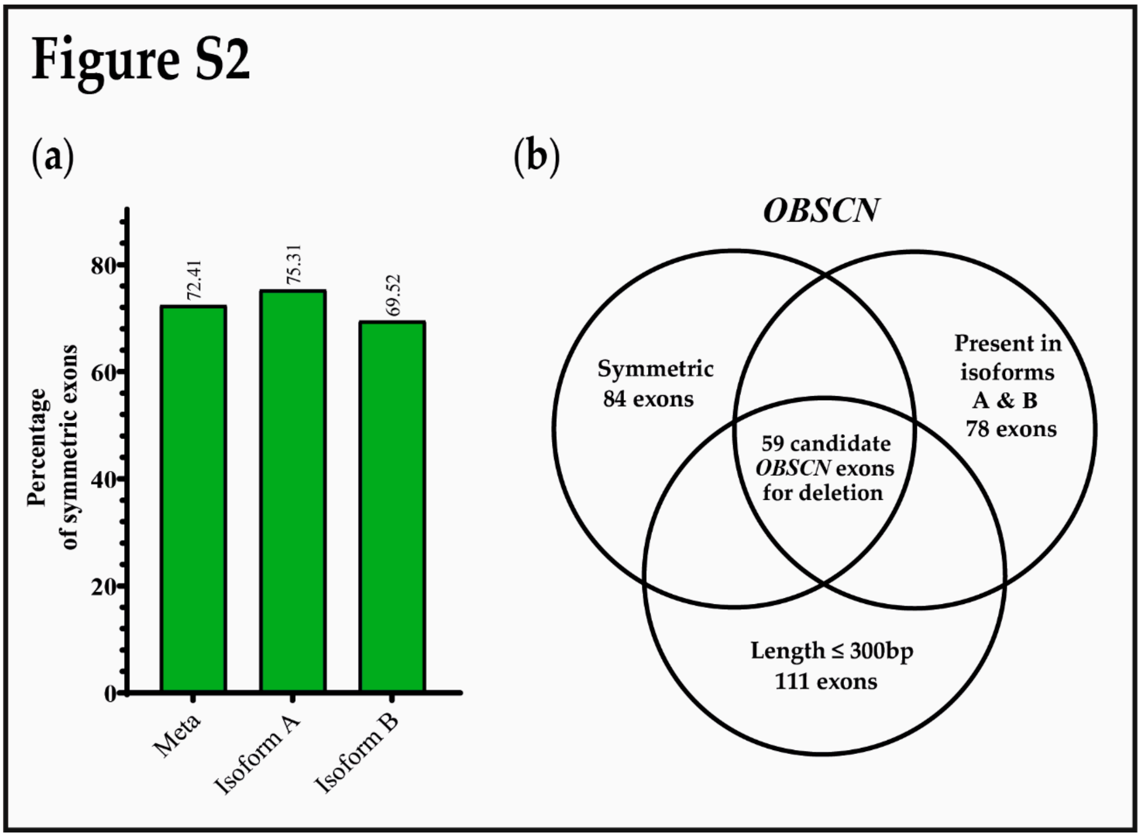

Figure S2. (**a**) Percentage of symmetric exons in Obscurin (*OBSCN*). Symmetric exons in the two major OBSCN isoforms (isoform A and B) and metatranscript (Meta). Accession numbers of the different sequences can be found in (**Table S4**). (**d**) Venn diagram of the parameters set for the identification of target exons. Selected exons are symmetric, present in isoforms A and B, and consist of maximally 300bp.

Table S5. Symmetric exon quantification in the major *OBSCN* isoforms (*Homo sapiens*).

Transcript	No. Exons*	Accession number	No. Symmetric exons	Percentage of symmetric exons (%)
Meta	116	ENST00000570156	84	72,41
Isoform A	81	ENST00000591111	61	75,31
Isoform B	105	ENST00000460472	73	69,52

*Number of protein-coding exons.

Table S6. List of candidate exons for removal. Exons were selected from the *OBSCN* metatranscript (ENST00000570156) and filtered according to symmetry, conservation between isoforms A and B (ENST00000591111 and ENST00000460472)(**Table S4**), and size (≤300bp). Additionally, domains and domain identifications (Pfam ID) are included in the table, together with a prediction of the number of interactions mediated by each domain (DI), predicted using DIGGER [51].

Exon Nº	Exon ID	Length	Domains	Exon Phase	Pfam ID	DI
**3**	ENSE00003729467	270	Ig-like	1	PF07679	5
**4**	ENSE00003751498	261		1	-	
**6**	ENSE00003732646	267		1	-	
**7**	ENSE00003628894	282	Ig-like	1	PF07679	5
**8**	ENSE00003488373	276	Ig-like	1	PF07679	5
**9**	ENSE00003380583	276	Ig-like	1	PF07679	5
**10**	ENSE00003734484	276	Ig-like	1	PF07679	5
**11**	ENSE00003734900	276	Ig-like	1	PF07679	5
**12**	ENSE00003753817	276	Ig-like	1	PF07679	5
**13**	ENSE00003744962	276	Ig-like	1	PF07679	5
**14**	ENSE00003720099	276	Ig-like	1	PF07679	5
**15**	ENSE00003735847	276	Ig-like	1	PF07679	5
**16**	ENSE00003723641	276	Ig-like	1	PF07679	5
**17**	ENSE00002358105	276	Ig-like	1	PF07679	5
**18**	ENSE00003714549	276	Ig-like	1	PF07679	5
**23**	ENSE00003712443	267		1	-	
**24**	ENSE00003752546	270	Ig-like	1	PF07679	5
**25**	ENSE00003712062	267	Ig-like	1	PF07679	5
**26**	ENSE00003751405	267		1	-	
**30**	ENSE00003722775	105	Ig-like	1	PF07679	5
**31**	ENSE00003724058	267	Ig-like	1	PF07679	5
**32**	ENSE00003739525	270		1	-	
**33**	ENSE00003716664	267	Ig-like	1	PF07679	5
**34**	ENSE00003721152	267	Ig-like	1	PF07679	5
**35**	ENSE00003467421	267	Ig-like	1	PF07679	5
**36**	ENSE00003517942	273	Ig-like	1	PF07679	5
**37**	ENSE00003714432	267	Ig-like	1	PF07679	5
**38**	ENSE00002353923	267	Ig-like	1	PF07679	5
**39**	ENSE00002336828	273	Ig-like	1	PF07679	5
**40**	ENSE00002423642	267	Ig-like	1	PF07679	5
**41**	ENSE00002358473	267	Ig-like	1	PF07679	5
**42**	ENSE00002424455	264	Ig-like	1	PF07679	5
**43**	ENSE00002379086	264	Ig-like	1	PF07679	5
**44**	ENSE00002339635	264	Ig-like	1	PF07679	5
**45**	ENSE00002394436	264	Ig-like	1	PF07679	5
**46**	ENSE00002398935	264	Ig-like	1	PF07679	5
**47**	ENSE00002424782	264	Ig-like	1	PF07679	5
**48**	ENSE00002356246	264	Ig-like	1	PF07679	5
**55**	ENSE00001069807	267	Ig-like	1	PF07679	5
**56**	ENSE00001069827	267	Ig-like	1	PF07679	5
**57**	ENSE00001069822	273	Ig-like	1	PF07679	5
**58**	ENSE00001069855	273	Ig-like	1	PF07679	5
**61**	ENSE00001069797	273		1	-	
**62**	ENSE00001069870	276		1	-	
**63**	ENSE00001069850	288	Fibronectin type-III	1	PF00041	3
**64**	ENSE00001069823	282	Ig-like	1	PF07679	5
**67**	ENSE00001069809	279	Ig-like	1	PF07679	5
**70**	ENSE00001069798	198	Ig-like	1	PF07679	5
**71**	ENSE00001069816	33		1	-	
**72**	ENSE00001069795	99	Ig-like	1	PF07679	5
**73**	ENSE00001069814	219	Ig-like	1	PF07679	5
**76**	ENSE00001069853	123		1	-	
**78**	ENSE00001069805	201		0	-	
**80**	ENSE00001069800	174	RhoGEF	2	PF00621	2
**82**	ENSE00001069801	117	RhoGEF	0	PF00621	2

**83**	ENSE00001069820	165	RhoGEF	0	PF00621	2
**84**	ENSE00001069842	159		0	-	
**86**	ENSE00001069840	114	Ig-like	1	PF07679	5
**87**	ENSE00001273763	168	Ig-like	1	PF07679	5
